# (S)-3-(3,4-Dihydroxybenzyl) piperazine-2,5-dione (cyclo-Gly-L-DOPA or CG-Nio-CGLD) peptide loaded in Chitosan Glutamate-Coated Niosomes as anti-Colorectal cancer activity

**DOI:** 10.1186/s40360-024-00766-2

**Published:** 2024-08-01

**Authors:** Tohid Piri-Gharaghie, Hedieh Ghourchian, Golnoosh Rezaeizadeh, Hamidreza Kabiri, Negin Rajaei, Aya Mohammed Dhiaa, Ghazal Ghajari, Roghayeh Bahari

**Affiliations:** 1 Biotechnology Research Center, Faculty of Biological Sciences, Shahrekord Branch, Islamic Azad University, Shahrekord, Iran; 2 Department of Biology, Faculty of Biological Science, Tehran North Branch, Islamic Azad University, Tehran, Iran; 3 Department of Microbiology, Faculty of Biological Sciences, Falavarjan Branch, Islamic Azad University, Isfahan, Iran; 4 Young Researchers and Elite Club, Shahrekord Branch, Islamic Azad University, Shahrekord, Iran; 5 Sina Borna Aria (SABA) Co., Ltd, Research and Development Center for Biotechnology, Shahrekord, Iran; 6 Department of Pharmacy, Al-Noor University College, Nineveh, Iraq; 7 Department of Biotechnology, College of Science, University of Tehran, Tehran, Iran; 8 Department of Biology, Faculty of Biological Science, Urmia Branch, Islamic Azad University, Urmia, Iran

**Keywords:** Cyclo-Gly-L-DOPA, Niosome nanoparticles, Cytotoxicity, Antiapoptotic activity

## Abstract

**Background:**

Colorectal cancer (CRC), now the second most prevalent malignant tumor worldwide, is more prevalent in young adults. In recent decades, there has been progress in creating anti-colorectal cancer medications, including cytotoxic compounds.

**Objectives:**

Novel anticancer drugs are needed to surmount existing obstacles. A recent study investigated the effectiveness of novel formulations in preventing colorectal cancer.

**Methods:**

During this study, we assessed a new kind of niosome called cyclo-Gly-L-DOPA (CG-Nio-CGLD) made from chitosan glutamate. We evaluated the anti-colorectal cancer properties of CG-Nio-CGLD utilizing CCK-8, invasion assay, MTT assay, flow cytometry, and cell cycle analysis. The transcription of genes associated with apoptosis was analyzed using quantitative real-time PCR. At the same time, the cytotoxicity of nanomaterials on both cancer and normal cell lines was assessed using MTT assays. Novel anticancer drugs are needed to surmount existing obstacles. A recent study investigated the effectiveness of newly developed formulations in preventing colorectal cancer.

**Results:**

The Nio-CGLD and CG-Nio-CGLD were spherical mean diameters of 169.12 ± 1.87 and 179.26 ± 2.17 nm, respectively. Entrapment efficiency (EE%) measurements of the Nio-CGLD and CG-Nio-CGLD were 63.12 ± 0.51 and 76.43 ± 0.34%, respectively. In the CG-Nio-CGLD group, the percentages of early, late, necrotic, and viable CL40 cells were 341.93%, 23.27%, 9.32%, and 25.48%. The transcription of the genes PP53, cas3, and cas8 was noticeably higher in the treatment group compared to the control group (*P* > 0.001). Additionally, the treatment group had lower BCL2 and survivin gene expression levels than the control group (*P* < 0.01). Additionally, CG-Nio-CGLD formulations demonstrated a biocompatible nanoscale delivery mechanism and displayed little cytotoxicity toward the CCD 841 CoN reference cell line.

**Conclusion:**

These findings indicate that chitosan-based noisome encapsulation may enhance the effectiveness of CG-Nio-CGLD formulations in fighting cancer.

## Introduction

Cancer is a prevalent global source of morbidity and mortality. Cancer ranks as the second most common cause of death among noncommunicable diseases, following cardiovascular disease [1]. Colorectal cancer (CRC) is the third most common kind of cancer and contributes significantly to cancer-related deaths worldwide [2]. The prevalence of colorectal cancer in the rectum and distal colon is 22% and 28%, respectively, accounting for approximately 41% of all occurrences of colorectal malignancies [3]. Several lifestyle factors that can raise the chance of getting colorectal cancer include excessive alcohol use, a diet high in fat and low in fiber, smoking, lack of physical activity, ageing, and having a family history of the disease [4]. Several modalities have been employed in cancer management, such as immunotherapy, chemotherapy, surgical intervention, and radiation therapy. Regrettably, the toxicity of these treatments can have detrimental effects on both healthy and cancerous cells, resulting in side effects such as nausea, gastrointestinal issues, reduced white blood cell levels, and hair loss [5, 6]. Scientists are investigating new chemical compounds that could specifically target and destroy cancer cells while minimizing harm to healthy cells [7, 8]. Cancer chemoprevention involves the utilization of organic or synthetic substances derived from diet or other sources to impede or decelerate the progression of cancer [8]. Chemoprevention is becoming increasingly popular since it can effectively decrease the occurrence of cancer-related diseases while causing minimal severe side effects [9]. Piperazine-2,5-dione and similar treatments can efficiently preserve numerous bioactive compounds that have potential as potent anticancer drugs [10].

Glycine anhydride, or piperazine-2,5-dione, is a cyclic peptide where oxo groups replace the hydrogen atoms at positions 2 and 5. It is a member of the 2,5-diketopiperazine category and is classified as a cyclic peptide. Piperazine-2,5-dione, a natural compound, is the smallest cyclic peptide and serves as a helpful framework, exhibiting diverse structural modifications [11]. Piperazine-2,5-diones demonstrate superior resistance to enzymatic degradation compared to their linear counterparts [12]. They also possess frequent conformational rigidity and can interact with various biological targets [5]. As a result, they exhibit a wide range of biological impacts [13], such as antiviral [14], anticancer [15], antifouling agents [16], antioxidative [17], and anti-PAI-1 properties [18], among others. Piperazine-2,5-dione has become a sought-after and unique framework for exploring potent pharmacological medicines. A specific group of piperazine-2,5-diones, which includes XR334 [19], piperazine B [20], phenylahistin [21], and its chemical derivative plinabulin [15–21], have unchanged C-C double bonds at the 3- and 6-positions of the Piperazine-2,5-dione ring. These chemicals demonstrate distinct and discernible variations in their ability to inhibit the growth of cancer cells. Piperazine-2,5-diones with phenyl ring at the 3- and 6-positions, such as piperazine B and XR334, will ultimately form linear and network structures due to the establishment of hydrogen bonding and π-stacking interactions between molecules [22].

Further biological research into these compounds has thus been halted because of their low lipo-solubilities and modest anticancer properties [21, 22]. However, those Piperazine-2,5-dione, including phenylahistin and plinabulin, that have imidazole molecules as an opposite section will have internal hydrogen links established more frequently among the amide bonding hydrogen of the Piperazine-2,5-dione ring and the nitrogen group of the opposite imidazole component [22], and the potential for the development of the intermolecular hydrogen links is vastly diminished. Their anticancer activity and lipo-solubilities have been significantly boosted [23]. By adding protecting groups to the amide nitrogen atom of the Piperazine-2,5-dione ring, analogous to the semi-N-methylation of piperafizine B to A, the lipophilicity of this type of molecule may also be increased. There is an increasing curiosity about innovative lipophilic items with a Piperazine-2,5-dione ring as their central component. In addition, the improved lipophilicity analysis of Piperazine-2,5-dione was advantageous to their anticancer properties. In our prior research, a single substance demonstrated beneficial anticancer effects on breast cancer cell lines [24].

Consequently, there is a growing fascination with creating innovative lipophilic substances with a central pillar composed of a diketopiperazine ring. The use of nano-systems for delivering medicinal substances to the targeted site of action is a stimulating and vital area of research in pharmacology [24]. Novel drug delivery strategies have significantly enhanced conventional medications’ therapeutic effectiveness and safety, particularly for compounds in nanoscale dimensions. In addition, they have the potential to provide regulated or sustained release routes to mitigate the advancement of adverse consequences [25]. Optimally, efficient delivery mechanisms are required to protect oral medications from the corrosive effects of stomach acid and enzymes. These techniques mitigate the negative impacts of food consumption on the absorption of medicine while enhancing the drug’s availability in the body. According to reports, chitosan and niosomes preferentially penetrate tumor cell membranes and exhibit anticancer action via cellular enzymatic, antiangiogenic, immune-stimulating, protective antioxidant processes, and apoptotic mechanisms. In addition, chitosan is a harmless, biocompatible natural substance [23–25]. The skeleton of arthropods, many crabs’ skins, fungus cell walls, and insects’ skin all contain chitosan, an organic and cationic polymer. Numerous research has shown the considerable pharmacological benefits of chitosan and its antimicrobial, anti-tumor, and antioxidant properties [25, 38]. Receptor-directed pharmaceutical targeting is a very active field of study that holds promise for developing innovative and effective therapies for many ailments. Considerable efforts have been dedicated to leveraging receptor-mediated transcytosis/endocytosis (RMT) as a means to improve cellular absorption in the area of medication delivery. Receptor-mediated targeting (RMT) refers to attaching targeting ligands, such as peptides, antibodies, or amino acids like Glutamate, to the ligands (protein biomarkers/antigens) found on the cell’s surface [25, 39, 40].

In addition, niosomes exhibit more excellent durability than liposomes and serve as effective delivery methods and non-toxic nanomaterials for therapeutic applications. Niosomes consist of non-ionic surfactants and have the potential to improve the penetration of substances into the cytoplasm. The distribution options for noisome/chitosan nanoparticles include intravenous, intramuscular, intrathecal, and epidermal routes. Studies have employed targeted, controlled, and sustained release methodologies in investigating different drug niosomal chitosan formulations [18, 20, 25]. Niosomal/chitosan formulations of piperazine-2,5-dione can enhance their ability to cross the blood-brain barrier and improve their pharmacokinetic properties when taken orally. They might be a promising treatment option for neurological indications and symptoms. Additional research has confirmed the anticancer characteristics of piperazine-2,5-dione, opening up a new field of investigation. This work introduces a unique formulation that combines piperazine-2,5-dione niosomes with chitosan encapsulation. Our research aims to thoroughly examine the medicinal chemistry, drug transport mechanism, and possible therapeutic applications of piperazine-2,5-dione, focusing on its impact on cancer cells.

## Materials and methods

### Materials

Cholesterol, diacetyl phosphate, Sephadex G75, pyrene, 1,6-diphenyl-1,3,5-hexatriene, the mucin from porcine intestinal type II substance, Tween 80 (polysorbate 80), chitosan medium molecular weight substance (1526.5 g/mol), and sodium hydroxide were bought from Sigma-Aldrich (Milan, Italy). PanReac Applichem (Milan, Italy) provided the L-glutamic acid. Every other item and substance were of analytical grade.

### Synthesis of (S)-3-(3,4-Dihydroxybenzyl) piperazine-2,5-dione (cyclo-Gly-L-DOPA or CGLD)

A mixture of (S)-Methyl 2-amino-3-(3,4-dihydroxyphenyl) propanoate hydrochloride (L-DOPA-OM) (4.02 g, 20.4 mmol) in ethanol (100 mL) was treated with thionyl chloride (13.5 mL, 184 mmol) at 0 °C. The solvent was removed after 21 h of room temperature use, and the residue was then concentrated to produce a light-yellow powder (5.01 g, 99%). L-DOPA-OM is the name of this yellow powder **(Composition 1).** A mixture of N, N-Dimethylformamide anhydrous (DMF, 15 mL), acetonitrile (60 mL), L-DOPA methyl ester hydrochloride (1.02 g, 4.11 mmol), and N-(tert-butoxycarbonyl)-glycine (0.72 g, 4.11 mmol) was chilled in ice. Triethylamine (0.5 mL, 3.6 mmol) and Dicyclohexyl-Carbodiimide (DCCI) (0.89 g, 4.33 mmol) were added while stirring. The solution was frozen overnight after 5 h of constant stirring at 0 °C. After being filtered out, the insoluble component was cleaned with ethyl acetate as the solvent. A sticky substance was left behind after the entire filtrate evaporated and dissolved in ethyl acetate (100 mL) and water (40 mL). Following each other, 40 mL volumes of 0.5 N HCl, H2O, 0.5 N NaHCO3, and brine were used to wash the organic layer. MgSO4 was used to dry the organic layer, and the filtrate solution was dehydrated. To get a colorless, amorphous powder (0.91 g, 60%), the remaining substance was submitted to silica columns of chromatography (ethyl acetate/hexane = 1:1, 3:2, then 2:1). This compound was (S)-Methyl 2-(2-((tert-butoxycarbonyl) amino) acetamido)-3-(3,4-dihydroxy phenyl) propanoate, which was abbreviated as (Boc-Gly-L-DOPA-OMe) **(Composition 2)**. 0.36 g of composition 2 (Boc-Gly-L-DOPA-OMe, 0.36 g, 0.97 mmol) was dissolved in 4 M HCl-dioxane (5 mL), the resulting solution was agitated for two hours at 25 °C, and then strained. The remaining material was dispersed in 12.5mL of 0.1 M AcOH-2-butanol after three co-evaporations with dioxane. Then, 0.09 mL of N-methylmorpholine was added. Three hours were spent refluxing the reaction solution. Colorless powder (0.12 g, 51%) was obtained by centrifuging an insoluble substance and washing it with ethanol after chilling at −20 °C. This compound was (S)-3-(3,4-Dihydroxybenzyl) piperazine-2,5-dione (cyclo-Gly-L-DOPA or CGLD) **(Final Composition)**.

### Synthesis and purification of niosomes

Thin film hydration was used to generate niosomes. Cholesterol (15 mM), tween 80 (7.5 mM), and diacetyl phosphate (7.5 mM) were submerged in a chloroform/methanol (3:1 v/v; 10 ml) organic chemical solvent combination. A thin “film” of the liquid was created using a rotary evaporator (VV2000, Heidolph, Germany). The ultrasonic microprobe (Vibra-Cell VCX-400, USA) was used to sonicate the film at 60 °C and 16% intensity for 5 min after hydrating it with 5 mL of HEPES solution (Sodium 2-(4-(2-hydroxyethyl) piperazin1-yl) ethanesulfonate) (0.01 M pH 7.4). The unilamellar vesicle solution was purified by gel extraction chromatography using Sephadex G75 (glass column of 50 × 1.2 cm) and HEPES solution as the solvent. In order to obtain accurate parameters and purify the niosome solution, the vesicles were passed through cellulose filters with the appropriate particle size.

### Preparation of chitosan glutamate-coated niosomes encapsulated cyclo-Gly-L-DOPA (CGLD)

Chitosan (1 mg) and glutamate (1 mg) were dissolved in acetate buffer (0.2 M, pH 4.4) to a final value of 0.05 mg/mL to generate a chitosan glutamate (CG) mixture. The resultant solution was agitated all night. Cyclo-Gly-L-DOPA (CGLD) loaded with niosomes was coated with CG by applying CG liquid in a 1:1 weight ratio to the various samples. In order to generate CG-coated niosomes (CG-Nio) and CG-coated niosomes with CGLD (CG-Nio-CGLD), the solution was stirred for 1 h at room temperature. An appropriate pH for oral administration has been confirmed for all formulations by evaluating the pH value (3.5 < pH < 6.4).

### **CG-Nio and CG-Nio-CGLD physicochemical characteristics**

The median dimensions, size distribution, and charge on the surface of CG-Nio and CG-Nio-CGLD were calculated utilizing dynamic light scattering (DLS) and ZetaPlas palladium substrates (Brookhaven Instruments Corp., USA). Several recently developed compositions were dissolved in purified water in a 20:1 proportion before the examination at 25 °C with a 90° light reflection angle to prevent double scattering caused by nanoparticle interactions. The median dimension and nanoparticle multiple scattering values were computed, and the zeta potentials of the nanomaterials were assessed. Investigations on CG-Nio and CG-Nio-CGLD were conducted using a field scanning electron microscope [FESEM] apparatus type MIRA3 [TESCAN, Czech Republic] coated with gold to give electrical conductivity.

### Entrapment efficiency [EE]

The effectiveness of entrapment (EE) of CGLD in the composition of Chitosan Glutamate-Coated niosomes was assessed using a Stat Fax2100 (Awareness Technology, Ukraine) ELISA Analyzer at 262 nm and determined through Eq. (1), using substances purified using gel filtration chromatography and purified on cellulose filters.

1 $$\left( {\text{\% }} \right){\text{EE}}=\frac{{{\text{Drug~detected~in~supernatant~}}\left( {{\text{mg}}} \right)}}{{{\text{Drug~added~}}\left( {{\text{mg}}} \right)}} \times 100$$

### Analysis of the vesicle bilayer

Characterization of the bilayers of CG-Nio and CG-Nio-CGLD has been done separately. Even though DPH and pyrene were both lipophilic compounds found within the bilayer, the probes’ data on the bilayer’s mobility, micro-viscosity, and polarity was different since they were studied using various fluorescence methods. Using both fluorescent probes may offer a complete view of the properties of the bilayer since DPH indicates lipid order and pyrene demonstrates horizontal diffusion within the bilayer.

Chloroform/methanol was used for Co-dissolved of tween 80 (7.5 mM), cholesterol (15 mM), DCP (7.5 mM), and DPH mixture (2 × 104 M). These components were then hydrated in HEPES solution (5 mg/mL), combined with a vortex mixer, and sonicated at 20 °C and 35% amplitude for five minutes. Following the solution’s filtering via a cellulose filter with a 450 nm cutoff, observations of its fluorescence spectrum (λ = 350–425 nm) were made using a luminescence analyzer (LS5013, PerkinElmer). Equation (2) was used to calculate the anisotropy of the fluorescence (r) [39].

2 $$Florenscence{\text{~}}Ansiotropy{\text{~}}\left( r \right)=\frac{{IVV{\text{~}} - {\text{~}}GIVH}}{{{\text{IVV~}}+{\text{~}}2{\text{GIVH}}}}$$

Where the fluorescence intensities IVV, IVH, IHV, and IHH are given, and the subscripts V (vertical) and H (horizontal) denote the direction of polarized light. The G factor measures a detecting system’s sensitivity to vertically and horizontally polarized radiation.

Pyrene-loaded niosomes were made by combining other vesicle components (4 mM pyrene) with them (same manufacturing process as previously). Fluorescence measurements may examine membrane molecules’ horizontal distribution and mobility. In contrast to excimer, which only has one peak (IE), the monomer of the fluorescent probe pyrene displayed five emission peaks in its spectrum (from I1 to I5). The monomer and the excimer produce different fluorescence signals, and the distribution of the probes inside the bilayer is directly correlated with the ratio between the various fluorescence intensities. The polarity of the probe context is precisely correlated with the ratio I1/I3, which corresponds to the initial and third vibrational bands in the pyrene spectrum. Pyrene has the potential to create intramolecular excimer depending on the viscosity of the probe microenvironment, which is calculated using the value of IE/I3, wherein IE is the excimer strength. Utilizing a luminescence analyzer (LS5013, PerkinElmer), the pyrene-loaded niosome suspension’s fluorescence spectra were scanned (λ = 350–550 nm), and the magnitudes of the excimer fluorescence (IE), initial (I1), and third (I3) peaks were noted.

### **Evaluation of CG-Nio and CG-Nio-CGLD release and stability**

Dialysis was used to evaluate the release of the CGLD through the chitosan glutamate-coated niosomes. The dialysis tube was immersed in purified water for 24 h. A volume of 0.5 ml [10 mg] of CG-Nio-CGLD was placed in the dialysis bag. Importantly, a control sample made out of CG-Nio water solution was used to ensure the validity of our results. Dialysis bags were placed inside conical flasks holding 75 ml of distilled water and agitated at 50 rpm in a tank of water at 37 °C. In order to measure CG-Nio employing spectrometers at 281 nm, 5 ml of the receptor media were taken out at periods of 0, 6, 9, 12, 18, 24, 30, 36, 48, 72, and 96 h. Aliquots of the samples were swapped out for new medium at 37 °C, and the diffusion pattern was determined using the kinetic model. Dynamic dialysis is a prevalent technique used to analyses the release kinetics from nanoparticle drug delivery systems. During two months of storage at 25 °C, the stability of the CG-Nio and CG-Nio-CGLD formulations was tested at periods of 0, 15, 30, 45, and 60 days.

### Cell culture

The Iranian Biological Resource Center (IBRC) provided the CL40 and SW1417 Colorectal cell lines, which were then cultivated by ATCC recommendations. Colorectal cell lines were grown in Dulbecco’s modified Eagle’s medium (DMEM; Gibco) at 37 °C and 5% CO_2_ and supplemented with 10% FBS, 50 µg/ml penicillin, and 50 µg/ml streptomycin (Sigma-Aldrich, USA).

### Analyze the colony growth and proliferation of CCK-8

The colon tumor cells in the experimental group (which had nanoparticle therapy) and the control group (which did not undergo nanoparticle treatment) were examined for CCK-8 using a CCK-8 detection kit from Dojindo, Japan. Following their implantation onto growth plates, the cells underwent nanoparticle treatment. The cell lines were incubated for 10 h before the CCK-8 chemical treatment was administered. The measurement of absorbance has been conducted at a wavelength of 450 nm.

### Transwell incursion detections

The adherent cells had already formed a layer on the surface of a 24-well transwell tube (Corning) (BD Biosciences, San Jose, CA, USA) before the targeted and control group of colorectal cancer cells were seeding. The invaded regions were treated with a 4% paraformaldehyde fixative and stained with Giemsa dye after a 24-hour treatment period. Subsequently, the upper surfaces were thoroughly cleaned. Subsequently, the cells were examined using an optical microscope.

### Cancer cell proliferation assay

Both the target group of colon tumor cells (those injected with nanoparticles) and the control group of colon tumor cells (those not treated with nanoparticles) had their cell viability confirmed with the MTT cell viability Kit I (Roche, Switzerland) colorimetric test. A 96-well flat-bottomed plate was seeded with 5 × 10^5^ cells per well, and the cells were grown there at 37 °C with 5% CO_2_. Over 24 h, the cells in each well were rinsed twice with PBS, and the vitality of the cells was evaluated. Each well received 100 µl of serum-free media with 5 µg/ml Sigma MTT before being placed in a CO_2_ incubator and maintained for 4 h at 37 °C. The medium was gradually taken out, and DMSO was added. The amount of optical density at 570 nm was contrasted with the background utilizing a State Fax-2100 ELISA plate scanner (Awareness Palm City, FL) to discover MTT metabolism that generates blue formazan.

### Analyzing the cell cycle using flow cytometry

A dual staining Annexin-V-FITC Propidium iodide binding test was used to measure the extent of cellular apoptosis by the manufacturer’s directions (Invitrogen TM, United Kingdom). The targeted cell line and control cells were grown on a 6-well plate at 106 cells/well. Following cell collection, three PBS washing cycles were completed. The mixed solution was centrifuged for five minutes at 400 g and then allowed to sit at 25 °C for 20 min. Samples have been dyed and inspected after being analyzed using the CyFlow ML flow cytometry technique (Partec, Germany). Cells from the control and study groups were fixed in pure ethanol for 24 h.

The cells are washed twice for 15 min in PI/RNase coloring reagent from BD Bioscience Pharmingen. The DNA of the cell population has been determined using FACS flow cytometry. The FlowJo V10 program (Tree Star, Ashland, OR) has evaluated the cell cycle information.

### Quantitative real-time PCR analysis of apoptosis-related gene

After the experiments, the data was meticulously analyzed to ensure the accuracy of our findings. A quantitative real-time PCR approach with SYBR green amplification was used to measure the transcription of the pro-apoptotic genes (*P53*, *cas3*, and *cas8*) and the anti-apoptotic genes *BCL2* and *SURVIVIN*. This was done using specific primers (Table 1). Cells were seeded at a density of 5 × 105 cells per well and treated for 72 h with IC50 nanoparticles before RNA was extracted. Total RNAs were extracted utilizing TRIZOL, and cDNA was generated using a kit (produced by Takara, Japan) according to the manufacturer’s instructions. Quantitative real-time PCR was carried out using the SYBR Green PCR kit for the apoptosis gene from Takara, Japan. The transcription of mRNA was assessed using the Qiagen Rotor-Gene Q kit. For the reverse transcription process, 1 µg of total RNA was taken from the specimens of each group. The SYBR Green Real-time PCR Master Mix (QuantiTec, Iran) and 0.3 mol of each primer pair were used in the qRT-PCR investigation to amplify the acquired cDNAs. Forty rounds of replication (94 °C for 15s, temperature of annealing for 30s, and 72 °C for 30s) followed a 5-minute initial qRT-PCR step at 94 °C. All experiments were done in triplicate, and the relative transcription of mRNA value to the control was recorded. The information has been adjusted to *GAPDH* mRNA.

### Cytotoxicity test using standard CCD 841 CoN cells

The cytotoxicity of CG-Nio and CG-Nio-CGLD has been evaluated using the MTT colorimetric assay (Kalazist, Iran). 5 × 10^5^ normal CCD 841 CoN cells have been grown in 96-well plates of DMEM with 10% fetal bovine serum. These plates have been placed in a CO2 incubator set at 37 °C. The cells have been exposed to CG-Nio and CG-Nio-CGLD at concentrations that ranged from 0.125 to 256 µg/mL. Following incubation, each well received 20 µL of the MTT mixture (Kalazist, Iran) (5 mg/mL in the colorless solution PBS). Following 4 h of incubation, the medium from the 96-well plates was removed, and 100 mL DMSO was injected. The formed formazan crystals in the DMSO were completely dissolved by spinning the plates at 400 rpm for six minutes. The standard deviation (SD) has been used to represent and depict the cell viability (*n* = 5). The colour intensity was assessed using an ELISA Scanner Stat Fax2100 at 570 nm. The fraction of viable cells was determined using the following equation:

3 $${\text{Cell~survival~rate\% }}=\frac{{{\text{~treated~cells~OD}}}}{{Control{\text{~}}cells{\text{~}}OD}} \times 100$$

**Table 1:** List of specific primers used in this research

Gene	Primer	Sequence 5′----------------------3′	TM (°C)
P53	P53-F	CCTCAGCATCTTATCCGAGTGG	59
	P53-R	TGGATGGTGGTACAGTCAGAGC	
Survivin	Sur-F	GAGAACGAGCCAGACTTGG	62
	Sur-R	GCTTTCCTTTCTGTCAAGAAGC	
BCL2	BCL2-F	TGTGGCCTTCTTTGAGTTCG	58
	BCL2-R	TACAGTTCCACAAAGGCATCC	
Cas3	Cas3-F	GGAAGCGAATCAATGGACTCTGG	59
	Cas3-R	GCATCGACATCTGTACCAGACC	
Cas8	Cas8-F	AGAAGAGGGTCATCCTGGGAGA	59
	Cas8-R	TCAGGACTTCCTTCAAGGCTGC	
GAPDH	GDH-F	GCCAAAAGGGTCATCATCTCTGC	62
	GDH-R	GCCAAAAGGGTCATCATCTCTGC	

### Statistical analysis

Each study was conducted in triplicate, and the data were presented as mean ± SD. The group differences have been investigated through one-way ANOVA and separate sample t-tests. SPSS software was used to analyze the data, and Graph-Pad Prism created the visualizations. The threshold for significance has been set at a *P* < 0.05.

## Results

### Synthesis (S)-3-(3,4-Dihydroxybenzyl) piperazine-2,5-dione (cyclo-Gly-L-DOPA or CGLD) as a drug

**Fig. 1:**
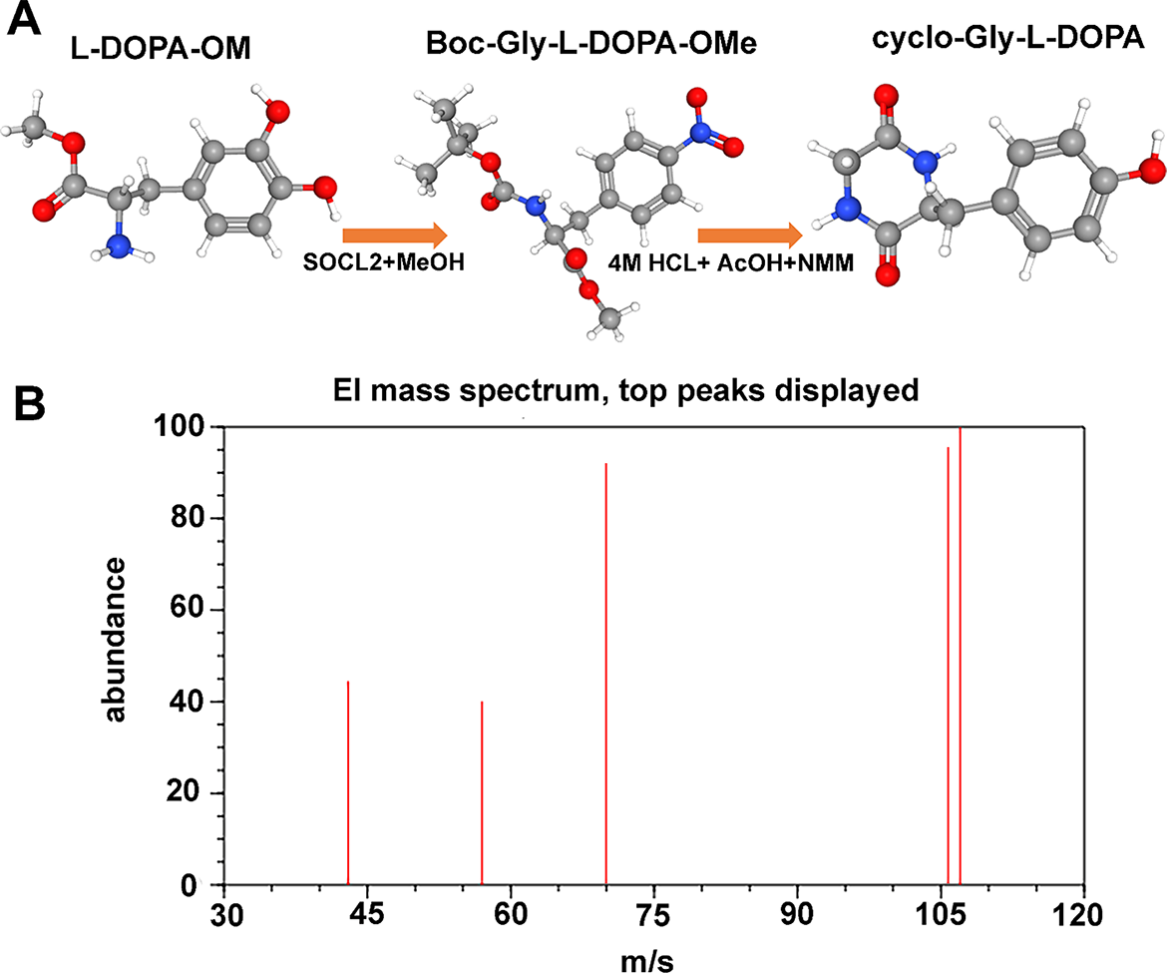
**A**) The blending of Boc-Gly and L-DOPA-OMe hydrochloride, followed by the creation of cyclo-Gly-L-DOPA are the steps in the process. **B**) The chart of GC-MS analysis of cyclo-Gly-L-DOPA

L-DOPA (**Composition 1**) is transformed to the equivalent methyl ester in ethanol at room temperature after a thionyl chloride reaction. In the existence of DCC in acetonitrile and DMF at 0 °C, Boc-Gly and L-DOPA-OMe hydrochloride were condensed. Utilizing silica column chromatography, the dipeptide was recovered. Boc-Gly-L-DOPA-OMe (**Composition 2**) underwent internal cyclization in a mixture of AcOH and N-methyl morpholine in 2-BuOH during reflux for several hours after Boc group deprotection. The precipitates were repeatedly washed with ethanol after the reaction solution became suspended to produce a modest yield of cyclo-Gly-L-DOPA (**Composition 3**). According to Fig. 1A, H NMR (DMSO-d6) of cyclo-Gly-L-DOPA was as follow: δ 2.68 (dd, *J* = 13.2, 7.6 Hz, 1 H), 2.92 (dd, *J* = 13.2, 3.5 Hz, 1 H), 3.38 (d, *J* = 16.8 Hz, 1 H), 3.50 (d, *J* = 16.8 Hz, 1 H), 4.20 (m, 1 H), 6.41 (d, *J* = 8.2 Hz, 1 H), 6.58 (d, *J* = 8.2 Hz, 1 H), 6.61 (s, 1 H); C NMR (DMSO-d6): 37.0, 40.7, 55.6, 115.4, 117.0, 120.0, 129.2, 143.7, 145.0, 166.2, 174.2; HRMS (ESI): calcd. for C11H13N2O4 (M + + H) 237.0875, found 237.0876; [α]D + 38.7 (c 2.0, DMSO). Also, the results of GC-MS analysis showed that this compound had 50 peaks, 107 m/z Top Peak, 43 m/z 2nd Highest and 57 m/z 3rd Highest. The chart of GC-MS analysis is shown in Fig. 1B. The usual lines of amine secondary stretching (-NH-) at 3400.39 cm^−1^, which intersect with hydroxy extending (-OH), were seen in the spectra. The amide carbonyl stretched [C(N) = O] is detectable at a wavelength of 1569.68 cm^−1^, which is less than the amide carbonyl stretched anticipated by DFT theory (1800 cm^−1^) because of hydrogen bonds between molecules in the natural molecule. A gentle peak at 1059.44 cm^−1^ (aromatic CH stretch) and prominent peaks at 956.21 cm^−1^ and 625.51 cm^−1^ (aromatic CC), 1414.01 cm^−1^ [(CO)], and 1031 cm^−1^ were further distinctive characteristics of the bands. The cyclo (Gly-L-DOPA) molecule, which has the chemical formula C11H12N2O3 and a molecular weight of 220.22 g/mol, has the properties listed in Table 2.

**Table 2:** Chemical and physical properties of cyclo-Gly-L-DOPA

Property name	Property value
Molecular Weight	220.22 g/mol
XLogP3-AA	0.3
Hydrogen Bond Donor Count	3
Hydrogen Bond Acceptor Count	3
Rotatable Bond Count	2
Exact Mass	220.08479225 g/mol
Monoisotopic Mass	220.08479225 g/mol
Topological Polar Surface Area	78.4 Å²
Heavy Atom Count	16
Formal Charge	0
Complexity	285
Isotope Atom Count	0
Defined Atom Stereocenter Count	1
Undefined Atom Stereocenter Count	0
Defined Bond Stereocenter Count	0
Undefined Bond Stereocenter Count	0
Covalently-Bonded Unit Count	1

### Morphology of the free noisome, CG-Nio, Nio-CGLD and CG-Nio-CGLD nanoparticles

Dynamic light scattering studies were used to determine nanoparticle materials’ size, -potential, and PDI. According to DLS and FeSEM, free niosome nanoparticles had median spherical sizes of 102.85 ± 3.04 and had average diameters of 159.16 ± 1.93 nm according to DLS (Table 3). Compared to the FeSEM assessment, the DLS test’s nanoparticle size was more significant. Hydrodynamic dimension is established via DLS. Therefore, the nanoparticles and the ions or compounds attached to the membrane are included in the measured dimensions. The incorporation of chitosan glutamate resulted in the coating of niosomes with a rise in zeta-potential levels to fewer negative ones, according to the data collected and shown in Table 3. The electrostatic relationship between positively charged chitosan and oppositely charged DCP on the outermost layer of the niosome bilayer causes the rise in zeta-potential. CG-Nio is tiny enough (117.32 ± 5.13 nm) to be delivered orally through the intra-intentional path, which requires a diameter of 1 micrometre (the luminal surface of the enterocytes microvilli) despite its modest size. The CG-Nio was chosen for further analysis because the Z-average stayed below 200 nm, and the PDI ratio (0.315 ± 0.04) remained acceptable at this nanopolymer.

**Fig. 2:**
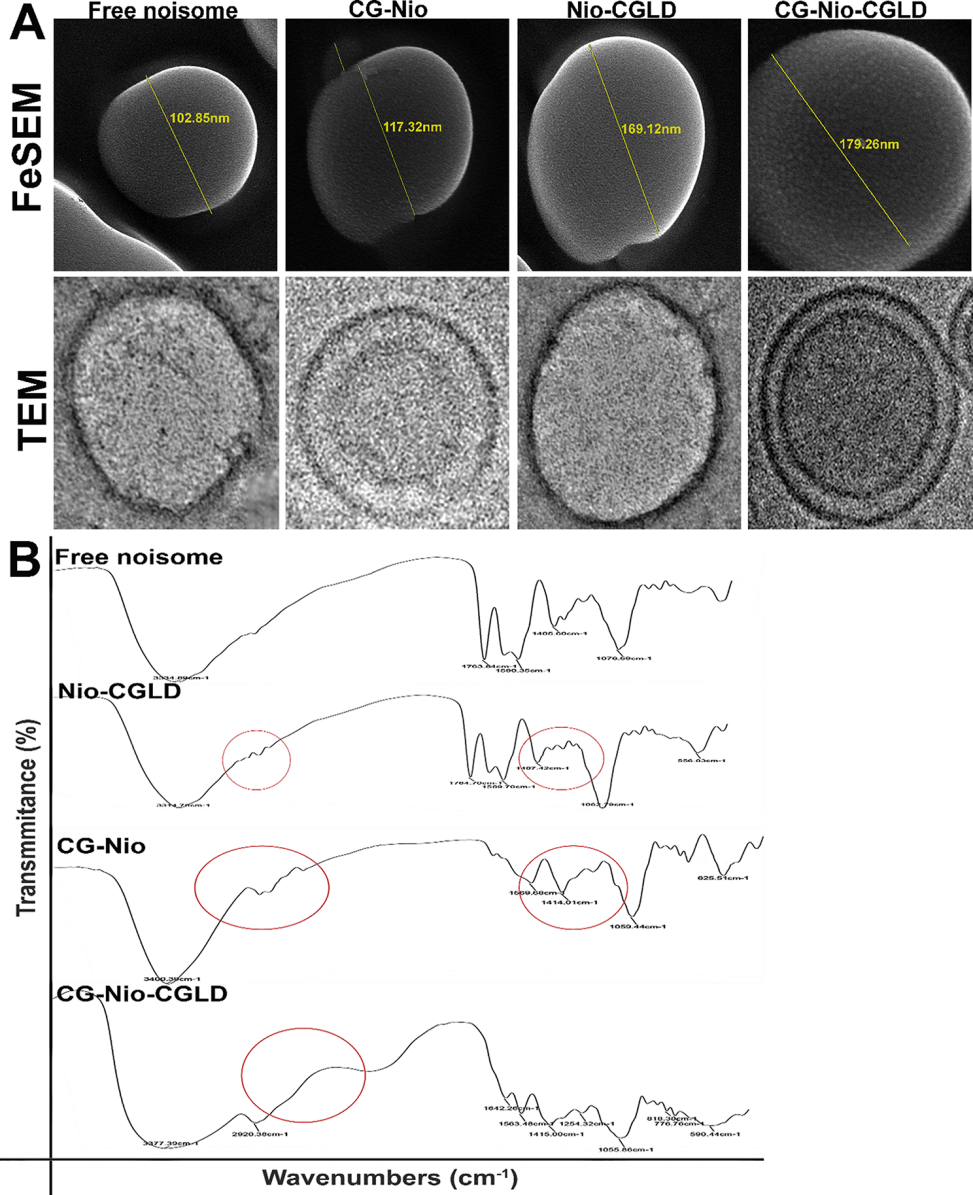
TEM, FeSEM electron microscopy images (**A**) and the FTIR spectra (**B**) of synthetic versions of Free noisome, CG-Nio, Nio-CGLD, and CG-Nio-CGLD. The images show the bilayer structure produced by the formulation of glutamate nanochitosan by Niosome

Furthermore, the zeta-potential of + 1.12 ± 1.23 mV validated the niosome coating, effectively inhibiting niosome agglomeration over time. Table 3 shows that the introduction of CGLD to the formulation resulted in an observable increase in vesicle diameter when comparing the dimensions of niosome and CG-Nio. The introduction of CG did not significantly increase the size of the niosome. Since the CGLD is present inside the bilayer and might have influenced the electrostatic interaction between chitosan and the bilayer, the increase in potential was somewhat lower compared to the niosome without any drugs. The PDI (Polydispersity Index) values of Nio-CGLD (0.214 ± 0.07) and CG-Nio-CGLD (0.196 ± 0.02), crucially below 0.300, indicate a narrow size distribution, providing a sense of reassurance. Additionally, the diameters of both particles were less than 200 nm. Figure 2A illustrates the characteristic topographical perspectives of CG-Nio, Nio-CGLD, and CG-Nio-CGLD, demonstrating their spherical shape and the absence of coalescence and relative uniformity in their size distribution.

**Table 3:** Characterization of free noisome, CG-Nio, Nio-CGLD and CG-Nio-CGLD

Nanomaterials	Polydispersity index (PDI)	Surface charge (mv)	Size (nm) (SEM)	Hydrodynamic diameter (nm)	EE (%)
Free niosome	0.415 ± 0.03	−16.19 ± 1.45	102.85 ± 3.04	159.16 ± 1.93	---
CG-Nio	0.315 ± 0.04	+ 1.12 ± 1.23	117.32 ± 5.13	178.20 ± 2.34	---
Nio-CGLD	0.214 ± 0.07	+ 3.23 ± 1.57	169.12 ± 1.87	192.3 ± 1.46	63.12 ± 0.51
CG-Nio-CGLD	0.196 ± 0.02	+ 4.78 ± 0.43	179.26 ± 2.17	198.9 ± 2.61	76.43 ± 0.34

Data are represented as mean ± SD, *n* = 3

### The FTIR spectra of formulations

Free noisome, CG-Nio, Nio-CGLD and CG-Nio-CGLD FTIR spectra are shown in Fig. 2B, and they demonstrate alterations, in particular of the individual features that resulted in the formation of CGLD. Broad indications at peak values at 3334.89 cm^−1^ and 1405.60 cm^−1^, which appear to represent C-H bond patterns, are shown by the free noisome (Fig. 2B). Peaks in the carboxyl group of the CG-Nio may be seen at 1059.44, 625.51 cm^−1^, whereas the amino group rises at 1414.01 cm^−1^. The Nio-CGLD’s FTIR spectrum (Fig. 2B) shows two CGLD peak values at 2920.38 cm^−1^ and 1254.32 cm^−1^ and two noisome peak levels at 1589.70 cm^−1^ and 1062.79 cm^−1^. The FTIR spectra of the CG-Nio-CGLD (Fig. 2B) indicate an increase at 3377.39 cm^−1^ and three CGLD peaks, identified by three irregular bending waves of the C-H in the aromatic ring of benzene at 818.38 cm^−1^, 2920.38 cm^−1^ and 1254.32 cm^−1^. The peak represents the O–H group at 1473.21 cm^−1^ in Fig. 2B.

### Encapsulation efficiency and in-vitro drug release

**Fig. 3:**
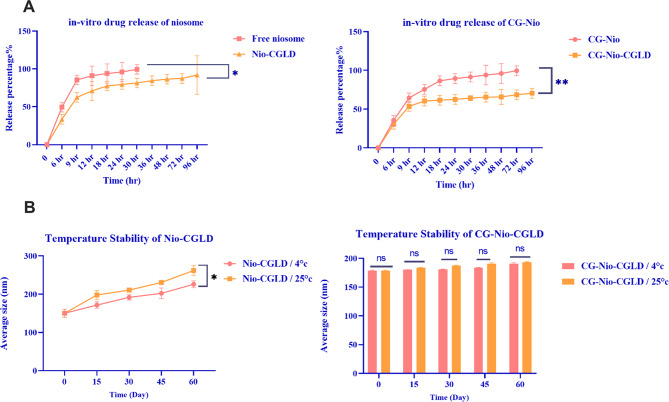
(**A**) Drug-Release examination; (**B**) impact of storage time and temperature on the median diameter of Nio-CGLD, and CG-Nio-CGLD. Data represent the average of three separate studies, plus or minus one standard deviation. **p < 0.01, *p < 0.05. *n* = 3

This study aimed to investigate the influence of the chitosan-glutamate combination on the formulation and release characteristics of Nio-CGLD. The drugs CG-Nio, Free Niosome, Nio-CGLD, and CG-Nio-CGLD were mixed in dialysis bags to investigate the mechanism of drug release. The entrapment efficiency (EE%) of Nio-CGLD and CG-Nio-CGLD was 63.12 ± 0.51% and 76.43 ± 0.34%, as shown in Table 3. The total diffusion rates of the Free niosome, CG-Nio, Nio-CGLD, and CG-Nio-CGLD were 86%, 65%, 63%, and 54%, respectively, after 9 h. Avoiding a lengthy release is advisable since it might diminish the drug’s efficacy and have adverse effects on the body. The CGLD contained inside the inner layers begins to release after nine hours after the quantity of CGLD in the outer layer has diminished. The diffusion rates of the Free niosome, CG-Nio, Nio-CGLD, and CG-Nio-CGLD were 100% (after 30 h), 100% (after 72 h), 94% (after 96 h), and 71% (after 96 h), respectively. Consequently, the pace at which medicine is released is slowed down. A rapid and explosive discharge of medicines marked the first hours. Based on this study, it was shown that Nio-CGLD, which is CGLD contained in niosomes, was forcefully evacuated. A consistent release pattern was observed for CG-Nio-CGLD (Fig. 3A). The interaction between the cationic lipid (CG) and the negatively charged vesicle (niosome) is a crucial factor in the sustained release of the medication.

### Analysis of the vesicle bilayer and stability test

The effect of chitosan glutamate on the niosome bilayer was investigated using fluorescent probes. The findings are shown in Table 4. Based on fluorescence anisotropy studies, it can be shown that the presence of chitosan glutamate (CG-Nio and CG-Nio-CGLD) does not significantly affect the fluidity of the bilayer, as compared to Nio and Nio-CGLD, respectively. The presence of the drug in Nio-CGLD and CG-Nio-CGLD led to lower levels, indicating that the drug causes the bilayer to be more fluid (with lateral motion inside the barrier) compared to niosomes without pharmaceuticals. The inverse relationship between fluorescence anisotropy levels and bilayer fluidity supports this.

**Table 4:** Characteristics of uncoated and CG-coated niosomes and Nio-CGLD bilayers

Sample	Fluidity (Anisotropy)	Microviscosity (IE/I3)	Polarity (I1/I3)
Free Niosome	0.27	0.73	1.09
CG-Nio	0.27	0.84	1.04
Nio-CGLD	0.19	0.23	1.18
CG-Nio-CGLD	0.14	0.21	1.17

To examine the stability of CG-Nio-CGLD at various temperatures and time levels, physicochemical characteristics, including the mean size, were evaluated. These findings imply that although encapsulating CGLD with niosome nanomaterials enhances drug stability, encapsulating CGLD with CG-Nio nanomaterials enhances the stability of the medicine that is discharged since CGLD showed more significant levels of stability after 60 days. It was demonstrated by comparing the median dimensions at different temperatures (4 °C and 25 °C) that increasing the temperature would change the morphological features of CGLD encapsulated in niosomes, decreasing the efficiency of encapsulation (Fig. 3B) but would not change the morphological features of CGLD encapsulated in CG-Nio (CG-Nio-CGLD).

### Malignant cell growth and invasion are inhibited by the CG-Nio-CGLD in vitro

**Fig. 4:**
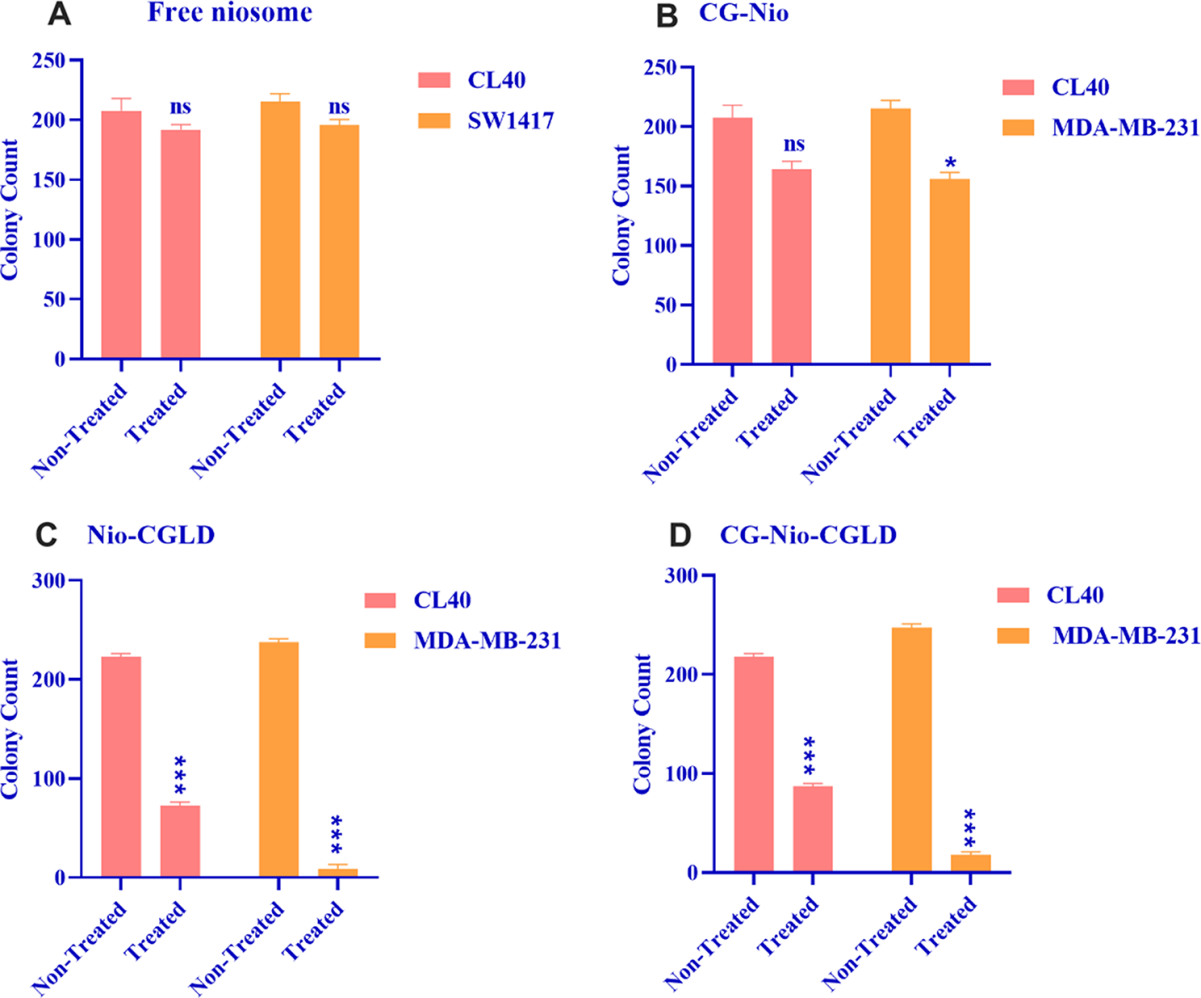
Malignant cell growth and invasion are inhibited in vitro by Nio-CGLD and CG-Nio-CGLD. In the CCK-8 test, it was shown that (**A**) free niosome, (**B**) CG-Nio, didn’t promoted cell proliferation but (**C**) Nio-CGLD and, (**D**) CG-Nio-CGLD promoted cell proliferation in CL40 and SW1417 cells

Colony formation assay and CCK-8 test findings showed that CGLD medicine reduced the growth of CL40 and SW1417 cells. Additionally, Nio-CGLD and CG-Nio-CGLD treatments reduced the proliferation of CL40 and SW1417 cells (*P* < 0.05 and *P* < 0.001, respectively). According to the transwell invasion experiment results, treatment of CL40 cells with Nio-CGLD and CG-Nio-CGLD decreased the number of invasive cancer cells by 38% and 62%, respectively. In contrast, there was no discernible alteration in the CG-Nio and Free Niosome groups. Also, treatment of SW1417 cells with Nio-CGLD and CG-Nio-CGLD decreased the number of invasive cancer cells by 42% and 66%, respectively. Therefore, the proliferation and invasion of these CL40, SW1417 cancerous cells were suppressed in vitro by being treated with Nio-CGLD and CG-Nio-CGLD (Fig. 4).

### CG-Nio-CGLD decreased cancer cell viability and didn’t have cytotoxicity for normal cells

**Fig. 5:**
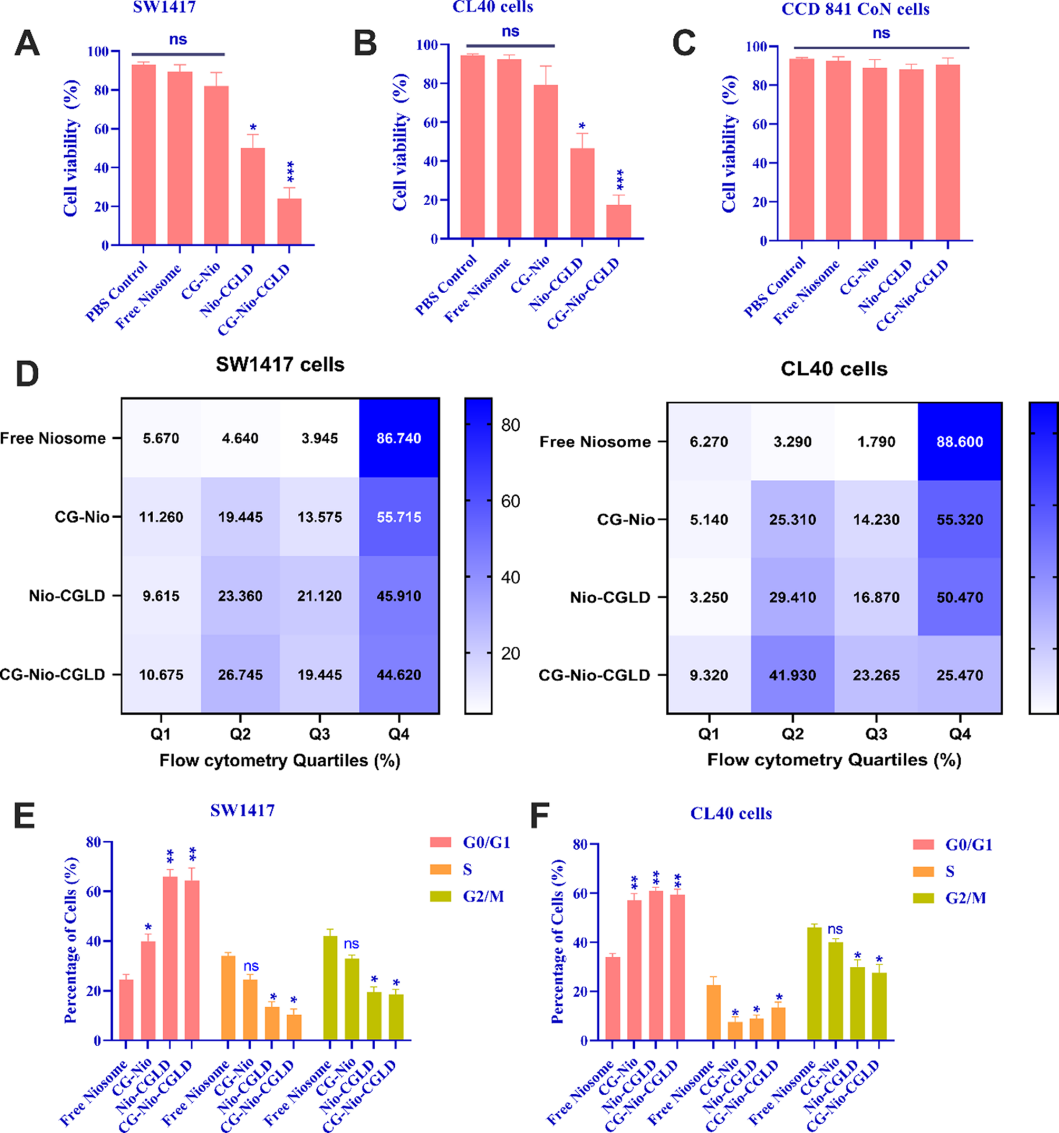
Free noisome, CG-Nio, Nio-CGLD and CG-Nio-CGLD dilute solutions on SW1417 (**A)**, CL40 (**B**), and CCD841 CoN (**C**) cell lines were examined for their effects on cell viability. (**D**) Flow cytometric investigation diagrams were shown in SW1417 and CL40 colorectal cancer cell lines. The cell cycle was regulated in SW1417 (**E**) and CL40 (**F**) colorectal cancer cell lines. Results are presented as mean ± SEM. The means with various superscript letters differ significantly (*P* ≤ 0.05)

MTT assessed how different formulations affected colorectal cancer cell viability at 24 h. The growth rates of the control and CG-Nio-CGLD groups differed significantly after 24 h (*P* < 0.001), and the growth percentage of colorectal cancer cells was lower in the CG-Nio-CGLD group. While the colorectal cancer cells in the CG-Nio and Free Niosome groups did not significantly differ in their rate of cell growth from the empty control (*P* > 0.05), the Nio-CGLD and CG-Nio-CGLD groups significantly decreased their rate of cellular growth within 24 h of treatment, with respective confidence levels of *P* < 0.05 and *P* < 0.001 (Fig. 5A and B). This discovery shows that the CG-Nio-CGLD has a significant detrimental impact on the growth of colorectal cancer cells.

Based on biocompatibility and biodegradability, chitosan and niosome are considered harmless. However, CGLD may be more toxic, depending on the formulation. The difference in the survival rate of healthy CCD 841 CoN cells among CG-Nio-CGLD and Nio-CGLD is seen in Fig. 5C. The findings indicate that CG-Nio-CGLD is not cytotoxic and could have increased the safety of free CGLD. According to earlier findings, CG-Nio-CGLD may boost cellular viability compared to Nio-CGLD. These results may be attributed to the fact that chitosan-glutamate-liposomal nano-drugs are mostly non-toxic to normal cells, and some investigations have shown that they can even lessen cytotoxicity.

### CG-Nio-CGLD increased apoptosis rate using flow cytometry

According to Fig. 5D, the cancerous cells in the treated group had more excellent apoptosis rates than those in the control group. Additionally, the cancer cell treatment group reported reduced cell viability. However, in the control groups, more than 90% of the cells were still alive, compared to fewer than 10% of early, late, and necrotic cells in the experimental groups. Early apoptosis, late apoptosis, necrotic, and alive CL40 cells were seen in 41.93%, 23.27%, 9.32%, and 25.48% of the cells with CG-Nio-CGLD, respectively. The percentage of SW1417 cells that underwent early apoptosis, late apoptosis, necrotic and alive cells was 26.75%, 19.45%, 10.68%, and 43.12%, respectively. Early apoptosis, late apoptosis, necrosis, and living cells were 29.41%, 16.87%, 3.25%, and 50.47% respectively, in the Nio-CGLD-treated CL40 cells. The percentages of early apoptosis, late apoptosis, necrotic, and live cells in the Nio-CGLD-treated SW1417 cells were 23.26%, 21.12%, 9.61%, and 45.91%, respectively.

Evaluation of cell cycles was carried out using flow cytometry. Sub-G1 increased in target cell lines (CG-Nio-CGLD) compared to CG-Nio and Free Niosome groups, delaying the entrance of cells into the G2 phase. Thus, cell growth decreased in this group. According to cell cycle analyses (Fig. 5E and F), CG-Nio-CGLD mostly halted the cell cycle in the G0/G1 phase.

### CG-Nio-CGLD enhanced apoptosis rate by suppressing anti-apoptotic genes

**Fig. 6:**
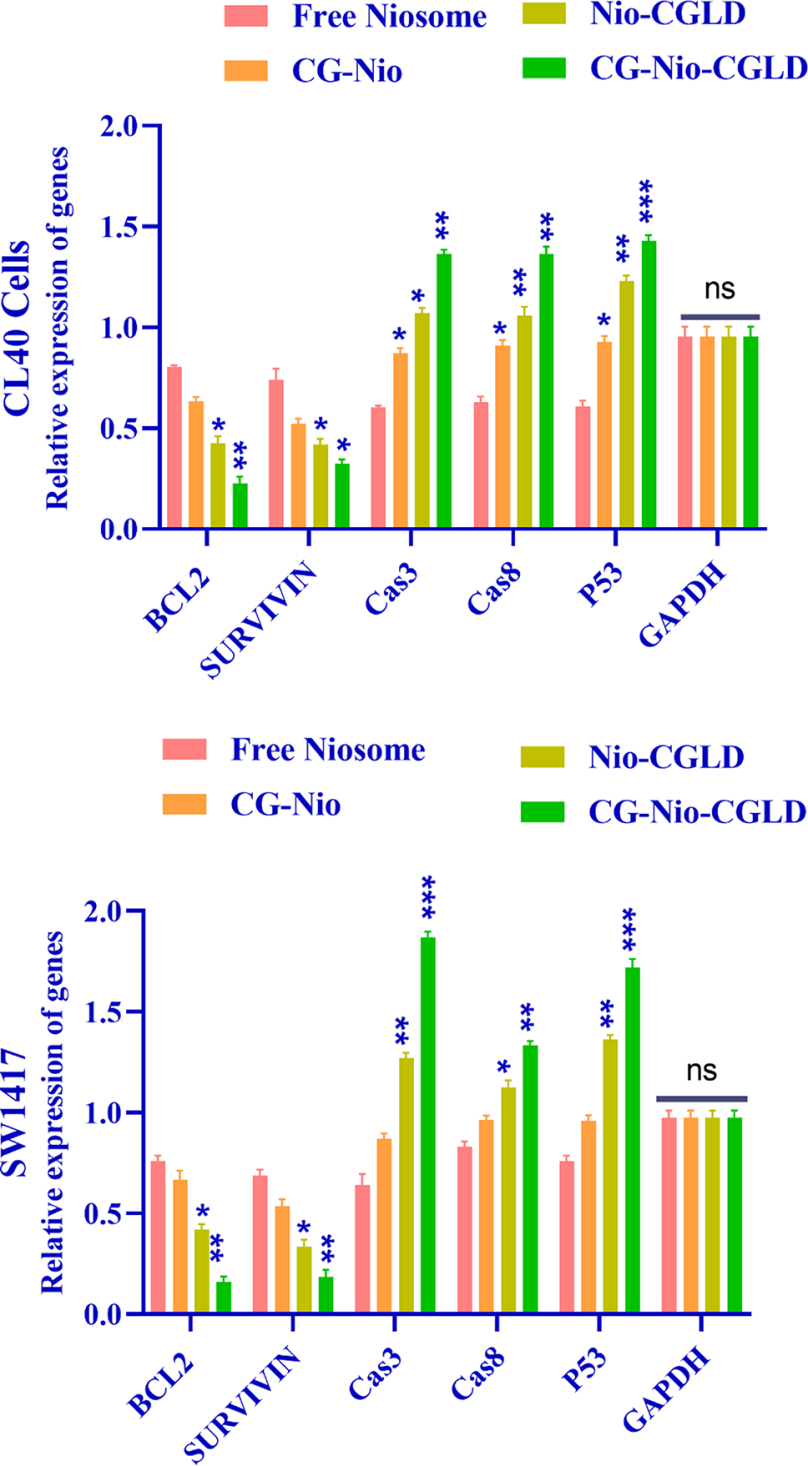
The effect of Nano-drug on cancer genes in CL40 and SW1417 cell line leads to induction of expression of pro-apoptotic genes *P53*,* cas3*, and *cas8* at a significant level ***P < 0.001 and reduction of expression of anti-apoptotic genes *SURVIVIN* and *BCL2* at a substantial level **P < 0.01. The GAPDH reference gene normalized data. There was no significant difference in the expression of pro/anti-apoptotic genes in the Blank control groups

Real-time PCR was used to analyze the transcription in CL40 and SW1417 distinct cell lines of the proapoptotic genes *P53*,* cas3*, and *cas8*, as well as the antiapoptotic genes *BCL2* and *SURVIVIN*. Compared to the free niosome and CG-Nio groups, proapoptotic transcription was significantly greater in the Nio-CGLD (*P* < 0.05) and CG-Nio-CGLD (*P* < 0.001) treatment groups. For the Nio-CGLD and CG-Nio-CGLD groups, respectively, significant rates were *P* < 0.05 and *P* < 0.001 (Fig. 6). We next examined the transcription of the *BCL2* and *SURVIVIN* antiapoptotic genes in two distinct cell types. *BCL2* and *SURVIVIN* gene transcription were more significant in the control cell line compared to the Nio-CGLD and CG-Nio-CGLD treated cells (*P* < 0.05 and *P* < 0.001, respectively).

## Discussion

This research aimed to investigate the impact of chitosan-glutamate niosome on the encapsulation and release characteristics of the CGLD medication. The study investigated the medicine release mechanism using various dialysis bags with various formulations. The Nio-CGLD and CG-Nio-CGLD exhibited entrapment efficiency (EE%) values of 63.12 ± 0.51 and 76.43 ± 0.34, respectively. Our achieved EE% percentage is much greater than what others have reported. A researcher in this field revealed the synthesis of drug-containing nanoparticles with an EE% ranging from 66.4 to 67.5% [26–28]. Following the rapid release of CGLD, the diffusion rates in Nio-CGLD and CG-Nio-CGLD reached 63% and 54%, respectively, during 9 h.

We have devised an innovative approach to enhance the energy efficiency (EE) of CGLD in Niosomes and achieve reduced values of size and PDI. Both chitosan and glutamate were dissolved in an acetate buffer to create a combination called chitosan glutamate (CG). The resulting solution was stirred vigorously during the whole night. The process included coating Cyclo-Gly-L-DOPA (CGLD) loaded with niosomes by adding CG liquid in a 1:1 ratio to the different samples. In order to create CG-coated niosomes (CG-Nio) and CG-coated niosomes with CGLD (CG-Nio-CGLD), the solution was stirred for 1 h at room temperature. The primary distinction of this approach, in comparison to others, is in the starting volume of the aqueous solution and the proportion of the organic phase to the aqueous phase. The findings align with existing research, which indicates that passive loading often leads to an encapsulation efficiency (EE) of around 1% for most medications. This low efficiency results in significant drug wastage [28, 41]. Drugs like CGLD often exhibit low efficiency of extraction (EE) because they tend to migrate quickly, resulting in the loss of the drug into the aqueous phase [26, 28, 41]. The poor efficacy of CGLD encapsulation is a consequence of the higher amount of the aqueous phase in the external environment of the niosomes compared to the restricted aqueous capacity inside the niosomes [26, 28, 41]. 1 H NMR analyses, used to assess the impact of extrusion on the reduction in the encapsulation efficiency (EE), indicate that the surface concentration of the cationic gemini lipid derivative (CGLD) in the niosomes also diminishes after extrusion. The application of pressure during this operation may result in an increased release of this CGLD, resulting in decreased EE.

It is recommended to avoid a prolonged release because it might reduce the drug’s potency and have a detrimental impact on the body [26–28]. The speedy first release of the drugs may first be attributed to them. As time goes on, more drug encapsulates are released through the surface. The encapsulated medications in the inner layers are released after nine hours when the quantity of CGLD on the surface is reduced. The rate of medication release is thus slowed down as a consequence. Previous studies found that the key factors affecting drug release from polymeric nanoparticles were biodegradability and diffusion. Rapid drug release within the first several hours was how the explosive release was characterized [26–28]. This experiment and earlier investigations showed that encapsulated CGLD was explosively released [27, 28]. The exchange of charges between the drug and the niosome, which occurs throughout the medication’s continuous-release, is essential [29].

During this trial, an initial occurrence of a rapid and sudden release of the medication was observed. The efficacy of this release modality surpasses that of the gradual release approach in cancer therapy. The first rapid release during the first 6 h may provide a potent and substantial medication dosage to the cancerous cell, inducing a state of shock and tension in the cell. Subsequently, by the sustained administration of the medication throughout the second stage, the proliferation and resistance of cancer cells are effectively inhibited. Thus, it may be inferred that the first abrupt discharge represents a crucial impact on the cancer cell, inducing a state of shock. Subsequently, the gradual and uninterrupted release during the second stage hampers the activity of cancer cells. Slow-release techniques include gradually releasing a certain medication dosage over a specific period. This might potentially lead to the development of drug resistance in cancer cells [29, 41, 42].

Other researchers concentrated on manufacturing factor optimization to create drug-loaded nanomaterials. The findings revealed that the drug-loaded nanoparticles had particle sizes ranging from 276 to 1034 nm [29, 30]. A researcher created the drug-loaded nanoparticles to fight dangerous microbes rather than infections. According to the SEM, the produced nanomaterials were 28.94 nm in size and almost spherical [31]. Another research [32] found that the produced nanomaterials had 273 hydrodynamic dimensions (Z average = 175 ± 21), which was equal to the diameter identified by TEM examinations (153 ± 42).

Prolonging the storage duration does not affect the morphology or efficacy of the drug’s encapsulation. An investigation was conducted to assess the stability of the synthetic medication using nanotechnology. The findings revealed that temperature significantly influences the disintegration of the drug [33]. The nanoparticles changed their morphological qualities, similar to when the temperature steadily increased over 60 days, decreasing their effectiveness. However, the results of this research indicate that nanostructure medications may be kept for 15 to 30 days without experiencing significant loss in their effectiveness. The morphological characteristics and efficacy of the CG-Nio-CGLD nanomedicine can be maintained for 60 days at a temperature of 25 °C, while Nio-CGLD can only be retained for a maximum of 60 days at a temperature of 4 °C. Previous research indicates that temperature also acts as a constraining element in the field of pharmacy [34]. As temperatures rise, the viscosity of fat increases, leading to an increase in the leakage of drugs. Consequently, the lipid complex known as niosome lacks stability when exposed to room temperature. However, by applying a coating of chitosan glutamate to the niosome, its stability at ambient temperature may be guaranteed [34, 35].

The MTT assay was used to evaluate the effect of CG-Nio-CGLD on the viability of colorectal cells after 24 h. The research showed a notable growth rate disparity between the control and CG-Nio-CGLD groups after 24 h (*P* < 0.001). The CG-Nio-CGLD group exhibited a reduced proliferation rate of colorectal cancer cells. The incidence of different treatment modalities in colorectal cancer cell groups did not exhibit significant differences compared to the control group (*P* > 0.05). In addition, the Nio-CGLD group exhibited a significant reduction in proliferation rate after 24 h of treatment, with a confidence level of *P* < 0.05. The data demonstrates that the CG-Nio-CGLD directly suppresses the proliferation of colorectal cancer cells.

Chitosan and niosome are regarded as safe because of their biocompatibility and biodegradability. However, the toxicity of CGLD might vary depending on its formulation. The results of this study illustrate the variation in the likelihood of survival of normal CCD 841 CoN cells when exposed to loaded CG-Nio, Nio-CGLD, CG-Nio-CGLD, and blank niosomes. The results suggest that CG-Nio-CGLD does not exhibit cytotoxicity and may enhance the safety profile of free CGLD. Previous research [35–36] shows that CG-Nio-drug can enhance cellular viability compared to free medicines. The findings may be ascribed to the exceptional non-toxicity of chitosan-based niosomal nano-drugs towards normal cells.

Furthermore, several studies have shown their ability to decrease cytotoxicity. Multiple studies have shown that using polymeric nanoparticles to encapsulate various drugs reduces their harmful effects on healthy cells [36, 37]. Research [37–40] found that the biocompatibility of nanoparticles significantly reduced the adverse effects of iron oxide nanoparticles. The research found that the cytotoxicity of CGLD was reduced when encapsulated inside chitosan-based niosomal nanoparticles.

Also, to minimize the harmful effects on the standard cell line CCD 841 CoN, we developed a formulation of chitosan with glutamate that has reduced cytotoxicity. Glutamate triggers distinct characteristics in the chitosan-based niosome formulation, allowing it to target cancer cells specifically. Glutamate plays a vital role in cancer and quickly dividing cells by directly contributing to the metabolic pathways discussed above [8, 41, 42]. Glutamate also affects the advancement of tumors by interacting with its receptors since several studies have shown the presence of glutamate receptors in various types of tumor cells [41–43].

## Conclusions

This work has examined the cytotoxic effectiveness of CGLD and its chitosan-based niosomal substance on both colon cancer and healthy normal cells. Methods such as real-time PCR, MTT assay, flow cytometry analysis, and spectroscopy were used. The chitosan-based niosomal formulation of CGLD exhibited cytotoxicity against malignant cell populations while sparing normal cells. The expression of pro-apoptotic genes dramatically increased in cells treated with the medication, whereas the expression of anti-apoptotic genes significantly reduced. Chitosan-based niosomal nanoparticles improve the anticancer effects of CGLD by facilitating the transport of drugs. The findings indicate that using chitosan-based niosomal in CGLD compositions might be a favorable strategy for enhancing the effectiveness of anticancer treatment.

## Data Availability

The data used to support the findings of this study are available from the corresponding author upon request.
